# Supplementary figures and images for: Hematological convergence between Mesozoic marine reptiles (Sauropterygia) and extant aquatic amniotes elucidates diving adaptations in plesiosaurs
Source: PeerJ. 2019 Nov 19;7:e8022. doi: 10.7717/peerj.8022 (PMC6873879; doi:10.7717/peerj.8022)

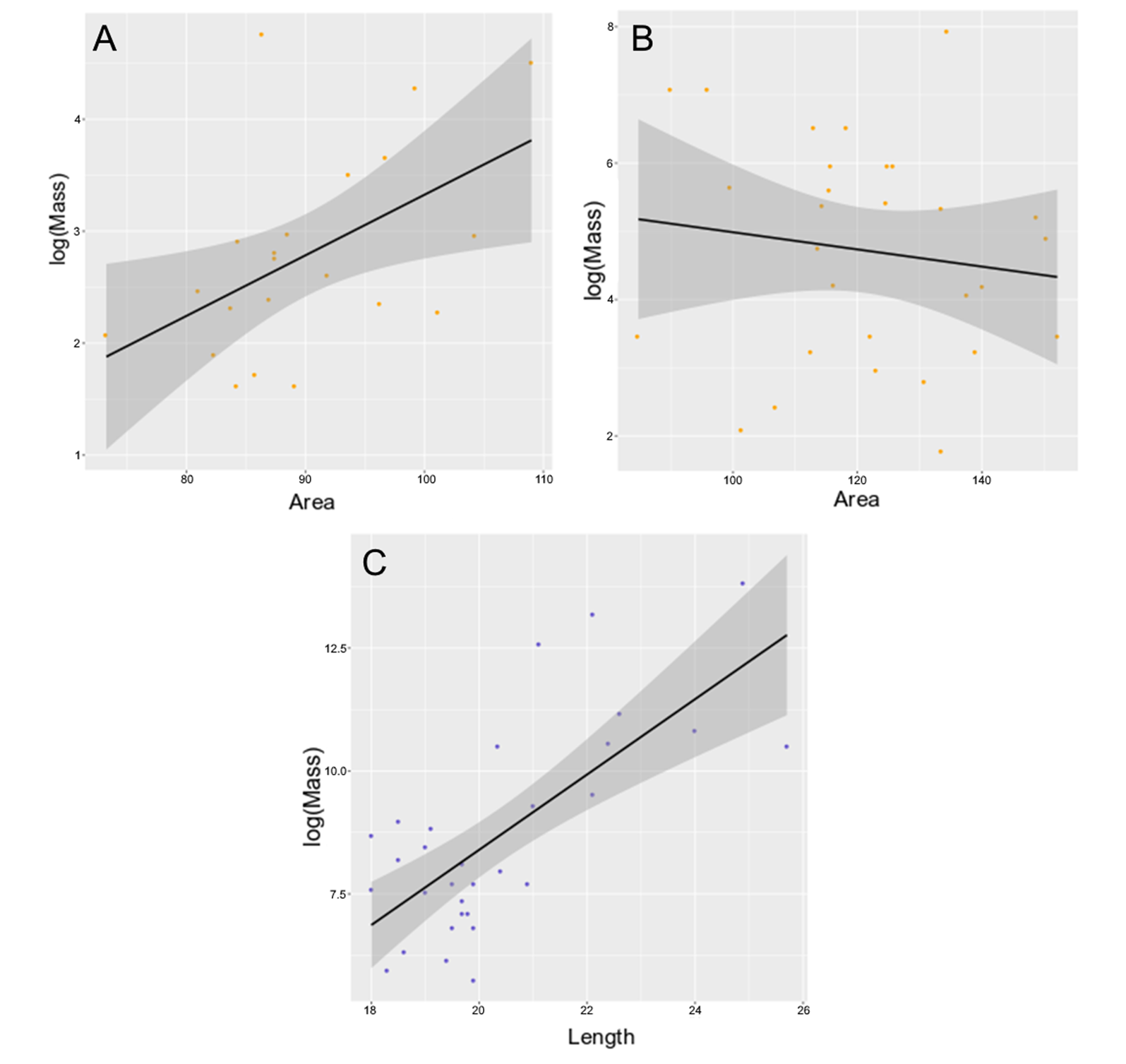

Supplement: Supplemental Information 1 — A: Lacertidae (n = 21, adjusted R2 = 0.218, p = 0.02). B: Colubridae (n = 42, adjusted R2 = −0.018, p = 0.48). C: Testudines (n = 31, adjusted R2= 0.488, p = 0.02). [file peerj-07-8022-s001.png]
