# Supplementary material for: Hematological convergence between Mesozoic marine reptiles (Sauropterygia) and extant aquatic amniotes elucidates diving adaptations in plesiosaurs
Source: PeerJ. 2019 Nov 19;7:e8022. doi: 10.7717/peerj.8022 (PMC6873879; doi:10.7717/peerj.8022)
Supplement: Supplemental Information 2 [file peerj-07-8022-s002.docx]

| **Taxon** | **Measured minimum vascular canal caliber (µm)** |
| --- | --- |
| *Anarosaurus heterodontus* | 7.4691 |
| *Neusticosaurus edwardsii* | 7.7539 |
| *Neusticosaurus peyeri* | 7.0000 |
| *Neusticosaurus pusillus* | 7.5000 |
| *Nothosaurus* sp. | 6.5100 |
| *Cymatosaurus* sp. | 8.7000 |
| *Pistosaurus longaevus* | 10.4839 |
| *Cryptoclidus eurymerus* | 10.4364 |
| Elasmosauridae indet. | 12.5397 |
| *Plesiosaurus dolichodeirus* | 12.7455 |
| *Pliosaurus* sp. | 16.2900 |
| *Polycotylus latipinnus* | 11.5400 |
| *Rhaeticosaurus mertensi* | 11.5435 |
