# Supplementary material for: Hematological convergence between Mesozoic marine reptiles (Sauropterygia) and extant aquatic amniotes elucidates diving adaptations in plesiosaurs
Source: PeerJ. 2019 Nov 19;7:e8022. doi: 10.7717/peerj.8022 (PMC6873879; doi:10.7717/peerj.8022)
Supplement: Supplemental Information 3 — t value refers to the t-rest of the respective line in the table. Pr(>|t|) is the p value for the t-test. V_1 is the selected vector of the model. The adjusted R2 of the model is 0.8799. The F statistic of the overall analysis is 48.6 on 2 and 11 degrees of freedom with a p value of 3.463e−06. The AICc of the model including minimum canal caliber as a predicting variable is 11.4738. The AICc of the model including phylogeny only is 16.1778. The steepness parameter was estimated to be a = 0.6408827. [file peerj-07-8022-s003.docx]

|  | **Estimate** | **Std. Error** | **t value** | **Pr(>\|t\|)** |
| --- | --- | --- | --- | --- |
| **Intercept** | 1.4440 | 0.5287 | 2.731 | 0.019527 |
| **Minimum_caliber** | 1.3214 | 0.2490 | 5.306 | 0.000250 |
| **V_1** | -1.4697 | 0.3293 | -4.464 | 0.000957 |
