# Supplementary material for: Hematological convergence between Mesozoic marine reptiles (Sauropterygia) and extant aquatic amniotes elucidates diving adaptations in plesiosaurs
Source: PeerJ. 2019 Nov 19;7:e8022. doi: 10.7717/peerj.8022 (PMC6873879; doi:10.7717/peerj.8022)
Supplement: Supplemental Information 4 — t value refers to the t-rest of the respective line in the table. Pr(>|t|) is the p value for the t-test. V_7, V_10 and V_1 are the selected vectors of the model. The adjusted R2 of the model is 0.8586. The F statistic of the overall analysis is 20.7 on 4 and 9 degrees of freedom with a p value of 0.0001452. The AICc of the model including minimum canal caliber as a predicting variable is −7.466441. The AICc of the model including phylogeny only is −8.148751. The steepness parameter was estimated to be a = 0.5479073. [file peerj-07-8022-s004.docx]

|  | **Estimate** | **Std. Error** | **t value** | **Pr(>\|t\|)** |
| --- | --- | --- | --- | --- |
| **Intercept** | 0.6951 | 0.2254 | 3.084 | 0.013050 |
| **Minimum_caliber** | 0.6623 | 0.1063 | 6.233 | 0.000153 |
| **V_7** | -0.3095 | 0.1170 | -2.644 | 0.026730 |
| **V_10** | -0.2666 | 0.1179 | -2.260 | 0.050151 |
| **V_1** | -0.2775 | 0.1366 | -2.031 | 0.072792 |
