# Supplementary material for: Hematological convergence between Mesozoic marine reptiles (Sauropterygia) and extant aquatic amniotes elucidates diving adaptations in plesiosaurs
Source: PeerJ. 2019 Nov 19;7:e8022. doi: 10.7717/peerj.8022 (PMC6873879; doi:10.7717/peerj.8022)
Supplement: Supplemental Information 5 [file peerj-07-8022-s005.docx]

|  | Lower | Mean RBC area | Upper |
| --- | --- | --- | --- |
| *Anarosaurus heterodontus* | 61.15 | 78.84 | 101.64 |
| *Neusticosaurus edwardsii* | 64.36 | 82.84 | 106.62 |
| *Neusticosaurus peyeri* | 54.83 | 72.36 | 95.51 |
| *Neusticosaurus pusillus* | 61.27 | 79.27 | 102.56 |
| *Nothosaurus* sp. | 48.89 | 65.75 | 88.41 |
| *Cymatosaurus* sp. | 76.62 | 96.45 | 121.41 |
| *Pistosaurus longaevus* | 97.24 | 123.41 | 156.62 |
| *Cryptoclidus eurymerus* | 94.14 | 122.66 | 159.82 |
| Elasmosauridae indet. | 114.54 | 156.35 | 213.42 |
| *Plesiosaurus dolichodeirus* | 117.68 | 159.76 | 216.87 |
| *Pliosaurus* sp. | 147.64 | 220.93 | 330.58 |
| *Polycotylus latipinnus* | 104.30 | 140.09 | 188.17 |
| *Rhaeticosaurus mertensi* | 106.29 | 140.16 | 184.81 |
