# Supplementary material for: Hematological convergence between Mesozoic marine reptiles (Sauropterygia) and extant aquatic amniotes elucidates diving adaptations in plesiosaurs
Source: PeerJ. 2019 Nov 19;7:e8022. doi: 10.7717/peerj.8022 (PMC6873879; doi:10.7717/peerj.8022)
Supplement: Supplemental Information 6 [file peerj-07-8022-s006.docx]

|  | Lower | Mean RBC width | Upper |
| --- | --- | --- | --- |
| *Anarosaurus heterodontus* | 7.16 | 7.99 | 8.91 |
| *Neusticosaurus edwardsii* | 7.34 | 8.19 | 9.14 |
| *Neusticosaurus peyeri* | 6.78 | 7.65 | 8.64 |
| *Neusticosaurus pusillus* | 7.16 | 8.01 | 8.96 |
| *Nothosaurus* sp. | 6.41 | 7.29 | 8.29 |
| *Cymatosaurus* sp. | 8.01 | 8.84 | 9.75 |
| *Pistosaurus longaevus* | 9.03 | 10.00 | 11.07 |
| *Cryptoclidus eurymerus* | 8.85 | 9.97 | 11.23 |
| Elasmosauridae indet. | 9.78 | 11.26 | 12.96 |
| *Plesiosaurus dolichodeirus* | 9.94 | 11.38 | 13.03 |
| *Pliosaurus* sp*.* | 11.20 | 13.39 | 16.00 |
| *Polycotylus latipinnus* | 9.31 | 10.66 | 12.20 |
| *Rhaeticosaurus mertensi* | 9.43 | 10.66 | 12.04 |
