# Supplementary material for: Hematological convergence between Mesozoic marine reptiles (Sauropterygia) and extant aquatic amniotes elucidates diving adaptations in plesiosaurs
Source: PeerJ. 2019 Nov 19;7:e8022. doi: 10.7717/peerj.8022 (PMC6873879; doi:10.7717/peerj.8022)
Supplement: Supplemental Information 7 — All measurements are given in µm. Names listed have been checked to comply with current taxonomic nomenclature and for this reason might deviate from the ones listed in the referenced literature. [file peerj-07-8022-s007.docx]

| **Taxon** | **Family** | | **Length** | **Width** | **Area** | **Source** |
| --- | --- | --- | --- | --- | --- | --- |
| *Ablepharus chernovi* | Scincidae | | 14.13 | N.A. | 84.12 | Arikan & Cicek, 2014 |
| *Acanthodactylus boskianus* | Lacertidae | | 14.22 | N.A. | 88.45 | Arikan & Cicek, 2014 |
| *Acanthodactylus erythrurus* | Lacertidae | | 13.6 | N.A. | 91.9 | Saint Girons, 1970 |
| *Acanthodactylus harranensis* | Lacertidae | | 15.46 | N.A. | 104.22 | Arikan & Cicek, 2014 |
| *Agama impalearis* | Agamidae | | 14.9 | N.A. | 96.1 | Saint Girons, 1970 |
| *Ameiva praesignis* | Teiidae | | 15 | N.A. | N.A. | Hartmann & Lessler, 1964 |
| *Anadia ocellata* | Gymnophthalmidae | | 16.3 | N.A. | N.A. | Hartmann & Lessler, 1964 |
| *Anatololacerta danfordi* | Lacertidae | | 14.14 | N.A. | 101.13 | Arikan & Cicek, 2014 |
| *Anolis biporcatus* | Dactyloidae | | 18.5 | N.A. | N.A. | Hartmann & Lessler, 1964 |
| *Anolis carolinensis* | Dactyloidae | | 14.8 | N.A. | N.A. | Hartmann & Lessler, 1964 |
| *Anolis carolinensis* | Dactyloidae | | 15.3 | N.A. | 106.9 | Saint Girons, 1970 |
| *Anolis pachypus* | Dactyloidae | | 15.6 | N.A. | N.A. | Hartmann & Lessler, 1964 |
| *Anolis polylepis* | Dactyloidae | | 18.4 | N.A. | N.A. | Hartmann & Lessler, 1964 |
| *Apalone spinifera* | Tryonychidae | | 19.8 | 13 | N.A. | Frair, 1977 |
| *Apalone spinifera* | Tryonychidae | | 22.3 | 12.5 | N.A. | Frair, 1977 |
| *Apathya cappadocica* | Lacertidae | | 13.42 | N.A. | 83.73 | Arikan & Cicek, 2014 |
| *Aspidoscelis sexlineata* | Teiidae | | 15.8 | N.A. | N.A. | Hartmann & Lessler, 1964 |
| *Atractaspis sp.* | Viperidae | | 18.5 | N.A. | 152.5 | Saint Girons, 1970 |
| *Atropoides nummifer* | Viperidae | | 23.4 | N.A. | N.A. | Hartmann & Lessler, 1964 |
| *Basiliscus basiliscus* | Corytophanidae | | 19.3 | N.A. | N.A. | Hartmann & Lessler, 1964 |
| *Basiliscus basiliscus* | Corytophanidae | | 18.6 | N.A. | N.A. | Hartmann & Lessler, 1964 |
| *Boa constrictor* | Boidae | | 17.97 | N.A. | N.A. | Sloboda et al., 2007 |
| *Boa constrictor* | Boidae | | 18.97 | N.A. | N.A. | Sloboda et al., 2007 |
| *Boaedon fuliginosus* | Lamprophiidae | | 16 | N.A. | N.A. | Sloboda et al., 2007 |
| *Bothriechis lateralis* | Viperidae | | 22.2 | N.A. | N.A. | Hartmann & Lessler, 1964 |
| *Bothrops atrox* | Viperidae | | 22.1 | N.A. | N.A. | Hartmann & Lessler, 1964 |
| *Bungarus fasciatus* | Elapidae | | 17.9 | N.A. | 133.4 | Saint Girons, 1970 |
| *Caiman crocodilus* | Alligatoridae | | 20.6 | N.A. | N.A. | Hartmann & Lessler, 1964 |
| *Caiman crocodilus* | Alligatoridae | | 16.9 | N.A. | 131.5 | Saint Girons, 1970 |
| *Caretta caretta* | Cheloniidae | | 22.1 | 13.4 | N.A. | Frair, 1977 |
| *Cerastes cerastes* | Viperidae | | 18.2 | N.A. | 134.1 | Saint Girons, 1970 |
| *Chalcides ocellatus* | Scincidae | | 14.68 | N.A. | 91.33 | Arikan & Cicek, 2014 |
| *Chamaeleo africanus* | Chamaeleonidae | | 17.6 | N.A. | 107.8 | Saint Girons, 1970 |
| *Chamaeleo chamaeleon* | Chamaeleonidae | | 15.97 | N.A. | 130.33 | Arikan & Cicek, 2014 |
| *Chelodina longicollis* | Chelidae | | 19 | 12.5 | N.A. | Frair, 1977 |
| *Chelodina longicolls* | Chelidae | | 21.8 | 12.8 | N.A. | Frair, 1977 |
| *Chelonia mydas* | Cheloniidae | | 21.1 | 12.7 | N.A. | Frair, 1977 |
| *Chelydra serpentina* | Chelydridae | | 22.6 | 12.5 | N.A. | Frair, 1977 |
| *Chelydra serpentina* | Chelydridae | | 21.6 | 12.8 | N.A. | Frair, 1977 |
| *Chelydra serpentina* | Chelydridae | | 22.1 | 14.8 | N.A. | Frair, 1977 |
| *Chrysemys picta* | Emydidae | | 18.1 | 11.3 | N.A. | Frair, 1977 |
| *Chrysemys picta* | Emydidae | | 20.9 | 12.3 | N.A. | Frair, 1977 |
| *Chrysemys picta marginata* | Emydidae | | 20.6 | 14.4 | N.A. | Frair, 1977 |
| *Chrysemys picta picta* | Emydidae | | 20.9 | 12.3 | N.A. | Frair, 1977 |
| *Clelia clelia* | Colubridae | | 20.2 | N.A. | N.A. | Hartmann & Lessler, 1964 |
| *Clemmys guttata* | Emydidae | | 18.3 | 12.5 | N.A. | Frair, 1977 |
| *Coleonyx brevis* | Eublepharidae | | 15.86 | N.A. | 117.03 | Starostova, Kratochvil & Frynta, 2005 |
| *Coleonyx elegans* | Eublepharidae | | 18.11 | N.A. | 141.8 | Starostova, Kratochvil & Frynta, 2005 |
| *Coleonyx mitratus* | Eublepharidae | | 18.16 | N.A. | 142.57 | Starostova, Kratochvil & Frynta, 2005 |
| *Coleonyx variegatus* | Eublepharidae | | 18.9 | N.A. | 142.5 | Saint Girons, 1970 |
| *Coleonyx variegatus* | Eublepharidae | | 17.69 | N.A. | 130.75 | Starostova, Kratochvil & Frynta, 2005 |
| *Coluber constrictor* | Colubridae | | 15.3 | N.A. | N.A. | Hartmann & Lessler, 1964 |
| *Coronella austriaca* | Colubridae | | 17.5 | N.A. | 140 | Saint Girons, 1970 |
| *Crocodylus niloticus* | Crocodylidae | | 16.4 | N.A. | 115.9 | Saint Girons, 1970 |
| *Crotalus durissus* | Viperidae | | 17.5 | N.A. | 134.6 | O'Dwyer, Moço & da Silva, 2004 |
| *Crotalus viridis* | Viperidae | | 17.9 | N.A. | 184.6 | Saint Girons, 1970 |
| *Crotaphytus collaris* | Crotaphytidae | | 17.8 | N.A. | 120 | Saint Girons, 1970 |
| *Ctenophorus reticulatus* | Agamidae | | 14.7 | N.A. | 92.4 | Saint Girons, 1970 |
| *Cyrtopodion heterocercum* | Gekkonidae | | 16.17 | N.A. | 111.77 | Arikan & Cicek, 2014 |
| *Cyrtopodion scabrum* | Gekkonidae | | 14.83 | N.A. | 97.13 | Arikan & Cicek, 2014 |
| *Darevskia praticola* | Lacertidae | | 13.08 | N.A. | 82.34 | Arikan & Cicek, 2014 |
| *Darevskia rudis* | Lacertidae | | 13.45 | N.A. | 87.46 | Sevinc, Ugurtas & Yildirimhan, 2000 |
| *Darevskia uzzelli* | Lacertidae | | 13.65 | N.A. | 84.22 | Arikan & Cicek, 2014 |
| *Darevskia valentini* | Lacertidae | | 13.32 | N.A. | 80.97 | Arikan & Cicek, 2014 |
| *Deirochelys reticularia* | Emydidae | | 19.5 | 11.7 | N.A. | Frair, 1977 |
| *Delma fraseri* | Pygopodidae | | 17 | N.A. | 129.8 | Saint Girons, 1970 |
| *Dendrophidion paucicarinatum* | Colubridae | | 17.1 | N.A. | N.A. | Hartmann & Lessler, 1964 |
| *Dermochelys coriacea* | Dermochelyidae | | 24.9 | 15.9 | N.A. | Frair, 1977 |
| *Diporiphora bilineata* | Agamidae | | 17.6 | N.A. | 124.6 | Saint Girons, 1970 |
| *Dipsosaurus dorsalis* | Iguanidae | | 18 | N.A. | 145.8 | Saint Girons, 1970 |
| *Dolichophis caspius* | Colubridae | | 14.91 | N.A. | 89.88 | Arikan & Cicek, 2014 |
| *Dolichophis jugularis* | Colubridae | | 16.29 | N.A. | 95.81 | Arikan & Cicek, 2014 |
| *Dolichophis schmidti* | Colubridae | | 16.21 | N.A. | 125.82 | Arikan & Cicek, 2014 |
| *Eirenis barani* | Colubridae | | 16.18 | N.A. | 122.98 | Arikan & Cicek, 2014 |
| *Eirenis coronella* | Colubridae | | 16.59 | N.A. | 133.52 | Arikan & Cicek, 2014 |
| *Eirenis decemlineatus* | Colubridae | | 14.75 | N.A. | 116.25 | Arikan & Cicek, 2014 |
| *Eirenis eiselti* | Colubridae | | 14.13 | N.A. | 106.84 | Arikan & Cicek, 2014 |
| *Eirenis levantinus* | Colubridae | | 16.6 | N.A. | 130.84 | Arikan & Cicek, 2014 |
| *Eirenis modestus* | Colubridae | | 14.47 | N.A. | 84.78 | Arikan & Cicek, 2014 |
| *Eirenis punctatolineatus* | Colubridae | | 16.22 | N.A. | 122.07 | Arikan & Cicek, 2014 |
| *Eirenis rothii* | Colubridae | | 14.77 | N.A. | 101.24 | Arikan & Cicek, 2014 |
| *Emys orbicularis* | Emydidae | | 19.99 | N.A. | 200.67 | Arikan & Cicek, 2014 |
| *Emys orbicularis* | Emydidae | | 19.9 | N.A. | 182.8 | Saint Girons, 1970 |
| *Emys orbicularis* | Emydidae | | 18 | 14.5 | N.A. | Frair, 1977 |
| *Emys orbicularis* | Emydidae | | 19.9 | 11.7 | N.A. | Frair, 1977 |
| *Emys orbicularis* | Emydidae | | 22 | 14.4 | N.A. | Frair, 1977 |
| *Eretmochelys imbricata* | Cheloniidae | | 22.6 | 14.8 | N.A. | Frair, 1977 |
| *Erythrolamprus bizona* | Dipsadidae | | 19.8 | N.A. | N.A. | Hartmann & Lessler, 1964 |
| *Erythrolamprus epinephelus* | Dipsadidae | | 16.9 | N.A. | N.A. | Hartmann & Lessler, 1964 |
| *Erythrolamprus taeniurus* | Dipsadidae | | 19.2 | N.A. | N.A. | Hartmann & Lessler, 1964 |
| *Eryx jaculus* | Boidae | | 16.36 | N.A. | 112.83 | Arikan & Cicek, 2014 |
| *Eublepharis angramainyu* | Eublepharidae | | 16.57 | N.A. | 116.29 | Arikan & Cicek, 2014 |
| *Eublepharis angramainyu* | Eublepharidae | | 20.85 | N.A. | 183.75 | Starostova, Kratochvil & Frynta, 2005 |
| *Eublepharis angramainyu* | Eublepharidae | | 20.76 | N.A. | 180.87 | Starostova, Kratochvil & Frynta, 2005 |
| *Eublepharis cf. fuscus* | Eublepharidae | | 19.01 | N.A. | 148.89 | Starostova, Kratochvil & Frynta, 2005 |
| *Eublepharis macularius* | Eublepharidae | | 19.75 | N.A. | 163.62 | Starostova, Kratochvil & Frynta, 2005 |
| *Eublepharius macularius* | Eublepharidae | | 19.69 | N.A. | 167.02 | Starostova, Kratochvil & Frynta, 2005 |
| *Eumeces schneideri* | Scincidae | | 15.17 | N.A. | 92.31 | Arikan & Cicek, 2014 |
| *Gehyra variegata* | Gekkonidae | | 17.2 | N.A. | 139.5 | Saint Girons, 1970 |
| *Glyptemys muhlenbergii* | Emydidae | | 21.6 | 13.6 | N.A. | Frair, 1977 |
| *Goniurosaurus araneus* | Eublepharidae | | 19.94 | N.A. | 159.51 | Starostova, Kratochvil & Frynta, 2005 |
| *Goniurosaurus luii* | Eublepharidae | | 19.53 | N.A. | 152.69 | Starostova, Kratochvil & Frynta, 2005 |
| *Gopherus polyphemus* | Testudinidae | | 19.1 | N.A. | N.A. | Hartmann & Lessler, 1964 |
| *Graptemys flavimaculata* | Emydidae | | 19.5 | 12.2 | N.A. | Frair, 1977 |
| *Graptemys oculifera* | Emydidae | | 19.7 | 11.9 | N.A. | Frair, 1977 |
| *Graptemys pulchra* | Emydidae | | 19.7 | 12.3 | N.A. | Frair, 1977 |
| *Hemidactylus turcicus* | Gekkonidae | | 16.56 | N.A. | 115.89 | Arikan & Cicek, 2014 |
| *Hemitheconyx caudicinctus* | Eublepharidae | | 19.35 | N.A. | 170.2 | Starostova, Kratochvil & Frynta, 2005 |
| *Hemorrhois nummifer* | Colubridae | | 15.61 | N.A. | 114.3 | Arikan & Cicek, 2014 |
| *Hemorrhois ravergieri* | Colubridae | | 14.76 | N.A. | 115.38 | Arikan & Cicek, 2014 |
| *Heteronotia binoei* | Gekkonidae | | 21.4 | N.A. | 179.9 | Saint Girons, 1970 |
| *Hierophis viridiflavus* | Colubridae | | 16.1 | N.A. | 124.8 | Saint Girons, 1970 |
| *Holodactylus africanus* | Eublepharidae | | 19.68 | N.A. | 158.44 | Starostova, Kratochvil & Frynta, 2005 |
| *Iguana iguana* | Iguanidae | | 15.4 | N.A. | N.A. | Hartmann & Lessler, 1964 |
| *Iguana iguana* | Iguanidae | | 15.3 | N.A. | 93.8 | Saint Girons, 1970 |
| *Kinosternon subrubrum* | Kinosternidae | | 19.9 | 12 | N.A. | Frair, 1977 |
| *Lacerta agilis* | Lacertidae | | 14 | N.A. | 93.6 | Saint Girons, 1970 |
| *Lacerta pamphylica* | Lacertidae | | 15.61 | N.A. | 96.77 | Arikan & Cicek, 2014 |
| *Lacerta trilineata* | Lacertidae | | 14.39 | N.A. | 86.31 | Arikan & Cicek, 2014 |
| *Lacerta viridis* | Lacertidae | | 14.94 | N.A. | 96.03 | Arikan & Cicek, 2014 |
| *Lacerta viridis* | Lacertidae | | 15.5 | N.A. | 125 | Saint Girons, 1970 |
| *Lampropeltis triangulum* | Colubridae | | 18.1 | N.A. | N.A. | Hartmann & Lessler, 1964 |
| *Laticauda colubrina* | Elapidae | | 19.2 | N.A. | 169.8 | Saint Girons, 1970 |
| *Leiolepis belliana* | Agamidae | | 15.35 | N.A. | 104.69 | Arikan & Cicek, 2014 |
| *Lepidochelys kempii* | Cheloniidae | | 22.4 | 14.7 | N.A. | Frair, 1977 |
| *Lepidochelys olivacea* | Cheloniidae | | 20.35 | N.A. | 194.28 | Arikan & Cicek, 2014 |
| *Lepidochelys olivacea* | Cheloniidae | | 25.7 | 14.4 | N.A. | Frair, 1977 |
| *Leptophis ahaetulla* | Colubridae | | 15.5 | N.A. | N.A. | Hartmann & Lessler, 1964 |
| *Lialis burtonis* | Pygopodidae | | 19.9 | N.A. | 193.5 | Saint Girons, 1970 |
| *Lycodryas sp.* | Lamprophiidae | | 16.6 | N.A. | 117.5 | Saint Girons, 1970 |
| *Macrochelys temminckii* | Chelydridae | | 24 | 15 | N.A. | Frair, 1977 |
| *Macroprotodon cucullatus* | Colubridae | | 16.4 | N.A. | 112.4 | Saint Girons, 1970 |
| *Macrovipera lebetina* | Viperidae | | 17.21 | N.A. | 133.11 | Arikan & Cicek, 2014 |
| *Malpolon insignitus* | Lamprophiidae | | 15.24 | N.A. | 133.6 | Arikan & Cicek, 2014 |
| *Masticophis flagellum* | Colubridae | | 16.5 | N.A. | N.A. | Hartmann & Lessler, 1964 |
| *Mastigodryas boddaerti* | Colubridae | | 16.3 | N.A. | N.A. | Hartmann & Lessler, 1964 |
| *Mauremys caspica* | Geoemydidae | | 18.99 | N.A. | 177.64 | Arikan & Cicek, 2014 |
| *Mauremys caspica* | Geoemydidae | | 19 | 10.9 | 162.5 | Saint Girons, 1970 |
| *Mauremys rivulata* | Geoemydidae | | 19.02 | N.A. | 182.74 | Arikan & Cicek, 2014 |
| *Mauremys rivulata* | Geoemydidae | | 24.5 | 13.1 | N.A. | Frair, 1977 |
| *Mesalina brevirostris* | Lacertidae | | 14.06 | N.A. | 89.09 | Arikan & Cicek, 2014 |
| *Montivipera albizona* | Viperidae | | 17.16 | N.A. | 130.72 | Arikan & Cicek, 2014 |
| *Montivipera wagneri* | Viperidae | | 17.63 | N.A. | 105.71 | Arikan & Cicek, 2014 |
| *Montivipera xanthina* | Viperidae | | 17.08 | N.A. | 96.78 | Arikan & Cicek, 2014 |
| *Myriopholis macrorhyncha* | Leptotyphlopidae | | 15.86 | N.A. | 115.75 | Arikan & Cicek, 2014 |
| *Naja kaouthia* | Elapidae | | 16.65 | N.A. | N.A. | Arikan & Cicek, 2014 |
| *Naja naja* | Elapidae | | 16.2 | N.A. | 114.5 | Saint Girons, 1970 |
| *Naja siamensis* | Elapidae | | 16.25 | N.A. | N.A. | Arikan & Cicek, 2014 |
| *Naja sumatrana* | Elapidae | | 16.7 | N.A. | N.A. | Arikan & Cicek, 2014 |
| *Natrix maura* | Colubridae | | 18.1 | N.A. | 150.4 | Saint Girons, 1970 |
| *Natrix natrix* | Colubridae | | 16.87 | N.A. | 134.46 | Arikan & Cicek, 2014 |
| *Natrix tesselata* | Colubridae | | 15.98 | N.A. | 99.61 | Arikan & Cicek, 2014 |
| *Nerodia sipedon* | Colubridae | | 18.2 | N.A. | N.A. | Hartmann & Lessler, 1964 |
| *Nerodia sipedon* | Colubridae | | 18.4 | N.A. | N.A. | Hartmann & Lessler, 1964 |
| *Nerodia taxispilota* | Colubridae | | 18.5 | N.A. | N.A. | Hartmann & Lessler, 1964 |
| *Opheodrys aestivus* | Colubridae | | 16.3 | N.A. | N.A. | Hartmann & Lessler, 1964 |
| *Ophiomorus punctatissimus* | Scincidae | | 15.14 | N.A. | 92.08 | Arikan & Cicek, 2014 |
| *Ophisops elegans* | Lacertidae | | 12.43 | N.A. | 73.27 | Arikan & Cicek, 2014 |
| *Oxyuranus scutellatus* | Elapidae | | 16.6 | N.A. | 118.7 | Saint Girons, 1970 |
| *Pantherophis guttatus* | Colubridae | | 18.9 | N.A. | N.A. | Hartmann & Lessler, 1964 |
| *Parvilacerta parva* | Lacertidae | | 13.63 | N.A. | 85.8 | Arikan & Cicek, 2014 |
| *Pelomedusa subrufa* | Pelomedusidae | | 19 | 10.2 | 150 | Saint Girons, 1970 |
| *Phrynosoma mcallii* | Phrynosomatidae | | 18.9 | N.A. | 149.8 | Saint Girons, 1970 |
| *Platyceps collaris* | Colubridae | | 14.4 | N.A. | 113.63 | Arikan & Cicek, 2014 |
| *Platyceps najadum* | Colubridae | | 15.47 | N.A. | 124.5 | Arikan & Cicek, 2014 |
| *Platyceps ventromaculatus* | Colubridae | | 15.94 | N.A. | 133.6 | Arikan & Cicek, 2014 |
| *Plestiodon fasciatus* | Scincidae | | 15.9 | N.A. | N.A. | Hartmann & Lessler, 1964 |
| *Podarcis muralis* | Lacertidae | | 13.93 | N.A. | 92.46 | Arikan & Cicek, 2014 |
| *Podarcis muralis* | Lacertidae | | 13.8 | N.A. | 86.9 | Saint Girons, 1970 |
| *Podarcis siculus* | Lacertidae | | 13.89 | N.A. | 87.41 | Arikan & Cicek, 2014 |
| *Psammobates geometricus* | Testudinidae | | 18 | 10 | N.A. | Frair, 1977 |
| *Psammodromus algirus* | Lacertidae | | 13.8 | N.A. | 84.3 | Saint Girons, 1970 |
| *Psammophilus blanfordanus* | Agamidae | | 12.83 | N.A. | 122.34 | Arikan & Cicek, 2014 |
| *Pseudechis australis* | Elapidae | | 18.4 | N.A. | 162 | Saint Girons, 1970 |
| *Pseudechis porphyriacus* | Elapidae | 17 | | N.A. | 146.8 | Saint Girons, 1970 |
| *Pseudemys concinna* | Emydidae | | 18.5 | 11.8 | N.A. | Frair, 1977 |
| *Python regius* | Pythonidae | | 18.03 | N.A. | N.A. | Sloboda et al., 2007 |
| *Rhinechis scalaris* | Colubridae | | 18.8 | N.A. | 115.8 | Saint Girons, 1970 |
| *Rhynchocalamus melanocephalus* | Colubridae | | 17.96 | N.A. | 138.88 | Arikan & Cicek, 2014 |
| *Sceloporus malachiticus* | Phrynosomatidae | | 16.9 | N.A. | N.A. | Hartmann & Lessler, 1964 |
| *Spalerosophis diadema* | Colubridae | | 15.74 | N.A. | 118.1 | Arikan & Cicek, 2014 |
| *Sphenodon punctatus* | Sphenodontidae | | 23.3 | N.A. | 252 | Saint Girons, 1970 |
| *Spilotes pullatus* | Colubridae | | 17.7 | N.A. | N.A. | Hartmann & Lessler, 1964 |
| *Stellagama stellio* | Agamidae | | 16.85 | N.A. | 120.71 | Arikan & Cicek, 2014 |
| *Sternotherus carinatus* | Kinosternidae | | 19.9 | 12.6 | N.A. | Frair, 1977 |
| *Sternotherus odoratus* | Kinosternidae | | 18.3 | 12.5 | N.A. | Frair, 1977 |
| *Sternotherus odoratus* | Kinosternidae | | 19.4 | 12.4 | N.A. | Frair, 1977 |
| *Sternotherus odoratus* | Kinosternidae | | 23 | 13.6 | N.A. | Frair, 1977 |
| *Suta suta* | Elapidae | | 16.8 | N.A. | 137.3 | Saint Girons, 1970 |
| *Telescopus fallax* | Colubridae | | 18.33 | N.A. | 148.8 | Arikan & Cicek, 2014 |
| *Telescopus nigriceps* | Colubridae | | 18.55 | N.A. | 152.14 | Arikan & Cicek, 2014 |
| *Terrapene carolina* | Emydidae | | 18.1 | 8.7 | N.A. | Frair, 1977 |
| *Terrapene carolina* | Emydidae | | 19.6 | 12.5 | N.A. | Frair, 1977 |
| *Terrapene carolina carolina* | Emydidae | | 19 | 9.9 | N.A. | Frair, 1977 |
| *Terrapene carolina carolina* | Emydidae | | 20.6 | 12.6 | N.A. | Frair, 1977 |
| *Terrapene carolina carolina* | Emydidae | | 21 | 12.1 | N.A. | Frair, 1977 |
| *Terrapene ornata* | Emydidae | | 18.6 | N.A. | N.A. | Hartmann & Lessler, 1964 |
| *Testudo graeca* | Testudinidae | | 17.35 | N.A. | 163.81 | Arikan & Cicek, 2014 |
| *Testudo graeca* | Testudinidae | | 18.5 | 10.6 | 153.8 | Saint Girons, 1970 |
| *Testudo graeca* | Testudinidae | | 21.2 | 12.5 | N.A. | Frair, 1977 |
| *Testudo graeca* | Testudinidae | | 20.4 | 12.6 | N.A. | Frair, 1977 |
| *Testudo graeca ibera* | Testudinidae | | 15 | 7.6 | N.A. | Frair, 1977 |
| *Thamnophis sauritus* | Colubridae | | 16.4 | N.A. | N.A. | Hartmann & Lessler, 1964 |
| *Timon princeps* | Lacertidae | | 14.98 | N.A. | 99.27 | Arikan & Cicek, 2014 |
| *Trachemys scripta* | Emydidae | | 19.5 | 11.6 | N.A. | Frair, 1977 |
| *Trachemys scripta elegans* | Emydidae | | 18.5 | 10.5 | N.A. | Frair, 1977 |
| *Trachemys scripta elegans* | Emydidae | | 17.4 | 9.9 | N.A. | Frair, 1977 |
| *Trachemys scripta ornata* | Emydidae | | 18.6 | 11.2 | N.A. | Frair, 1977 |
| *Trachemys scripta troosti* | Emydidae | | 20.1 | 10.6 | N.A. | Frair, 1977 |
| *Trachylepis aurata* | Scincidae | | 14.27 | N.A. | 84.88 | Arikan & Cicek, 2014 |
| *Trachylepis vittata* | Scincidae | | 14.14 | N.A. | 83.77 | Arikan & Cicek, 2014 |
| *Trapelus lessonae* | Agamidae | | 14.75 | N.A. | 100.78 | Arikan & Cicek, 2014 |
| *Uma inornata* | Phrynosomatidae | | 17.8 | N.A. | 128.5 | Saint Girons, 1970 |
| *Urosaurus graciosus* | Phrynosomatidae | | 14.7 | N.A. | 103.6 | Saint Girons, 1970 |
| *Varanus exanthematicus* | Varanidae | | N.A. | N.A. | 144.7 | Frýdlová et al., 2013 |
| *Varanus griseus* | Varanidae | | 16.24 | N.A. | 130.33 | Arikan & Cicek, 2014 |
| *Varanus indicus* | Varanidae | | N.A. | N.A. | 136.6 | Frýdlová et al., 2013 |
| *Varanus komodoensis* | Varanidae | | 24.45 | N.A. | N.A. | Arikan & Cicek, 2014 |
| *Vipera aspis* | Viperidae | | N.A. | N.A. | 154.5 | Saint Girons, 1970 |
| *Vipera berus* | Viperidae | | 16.1 | N.A. | 132.8 | Saint Girons, 1970 |
| *Vipera eriwanensis* | Viperidae | | 16.98 | N.A. | 101.16 | Arikan & Cicek, 2014 |
| *Walterinnesia morgani* | Elapidae | | 16.2 | N.A. | 129.12 | Arikan & Cicek, 2014 |
| *Xerotyphlops vermicularis* | Typhlopidae | | 16.57 | N.A. | 118.76 | Arikan & Cicek, 2014 |
| *Zamenis hohenackeri* | Colubridae | | 17.66 | N.A. | 137.55 | Arikan & Cicek, 2014 |
| *Zamenis longissimus* | Colubridae | | 12.71 | N.A. | 73.83 | Arikan & Cicek, 2014 |
| *Zamenis longissimus* | Colubridae | | 18.3 | N.A. | 152.1 | Saint Girons, 1970 |
| *Zootoca vivipara* | Lacertidae | | 14.1 | N.A. | 96.3 | Saint Girons, 1970 |
