# Supplementary material for: Hematological convergence between Mesozoic marine reptiles (Sauropterygia) and extant aquatic amniotes elucidates diving adaptations in plesiosaurs
Source: PeerJ. 2019 Nov 19;7:e8022. doi: 10.7717/peerj.8022 (PMC6873879; doi:10.7717/peerj.8022)
Supplement: Supplemental Information 8 — Names listed have been checked to comply with current taxonomic nomenclature and for this reason might deviate from the ones listed in the referenced literature. [file peerj-07-8022-s008.docx]

| **Taxon** | **Family** | **Mass** | **Source** |
| --- | --- | --- | --- |
| *Ablepharus chernovi* | Scincidae | 2.9 | Feldman et al., 2016 |
| *Acanthodactylus boskianus* | Lacertidae | 19.6 | Feldman et al., 2016 |
| *Acanthodactylus erythrurus* | Lacertidae | 13.7 | Feldman et al., 2016 |
| *Acanthodactylus harranensis* | Lacertidae | 19.5 | Feldman et al., 2016 |
| *Agama impalearis* | Agamidae | 82.9 | Feldman et al., 2016 |
| *Ameiva praesignis* | Teiidae | 470.1 | Feldman et al., 2016 |
| *Anadia ocellata* | Gymnophthalmidae | 10.3 | Feldman et al., 2016 |
| *Anatololacerta danfordi* | Lacertidae | 9.8 | Feldman et al., 2016 |
| *Anolis biporcatus* | Dactyloidae | 30.8 | Feldman et al., 2016 |
| *Anolis carolinensis* | Dactyloidae | 8.7 | Feldman et al., 2016 |
| *Anolis pachypus* | Dactyloidae | 3.3 | Feldman et al., 2016 |
| *Anolis polylepis* | Dactyloidae | 4.4 | Feldman et al., 2016 |
| *Apalone spinifera* | Tryonychidae | 10919.59 | Hone et al., 2013 |
| *Apathya cappadocica* | Lacertidae | 10.2 | Feldman et al., 2016 |
| *Aspidoscelis sexlineata* | Teiidae | 22.2 | Feldman et al., 2016 |
| *Atractaspis sp.* | Viperidae | N.A. |  |
| *Atropoides nummifer* | Viperidae | 347.5 | Feldman et al., 2016 |
| *Basiliscus basiliscus* | Corytophanidae | 554 | Feldman et al., 2016 |
| *Boa constrictor* | Boidae | 35283 | Feldman et al., 2016 |
| *Boaedon fuliginosus* | Lamprophiidae | 315.1 | Feldman et al., 2016 |
| *Bothriechis lateralis* | Viperidae | 521.2 | Feldman et al., 2016 |
| *Bothrops atrox* | Viperidae | 1803.4 | Feldman et al., 2016 |
| *Bungarus fasciatus* | Elapidae | 1777.1 | Feldman et al., 2016 |
| *Caiman crocodilus* | Alligatoridae | 57656.97 | Hone et al., 2013 |
| *Caretta caretta* | Cheloniidae | 545000 | Duermit, 2007 |
| *Cerastes cerastes* | Viperidae | 371.3 | Feldman et al., 2016 |
| *Chalcides ocellatus* | Scincidae | 79.7 | Feldman et al., 2016 |
| *Chamaeleo africanus* | Chamaeleonidae | 147.8 | Feldman et al., 2016 |
| *Chamaeleo chamaeleon* | Chamaeleonidae | 95.6 | Feldman et al., 2016 |
| *Chelodina longicollis* | Chelidae | 2903.15 | Hone et al., 2013 |
| *Chelonia mydas* | Cheloniidae | 293686.89 | Hone et al., 2013 |
| *Chelydra serpentina* | Chelydridae | 13730.68 | Hone et al., 2013 |
| *Chrysemys picta* | Emydidae | 2246.59 | Hone et al., 2013 |
| *Clelia clelia* | Colubridae | 1722.6 | Feldman et al., 2016 |
| *Clemmys guttata* | Emydidae | 386.88 | Hone et al., 2013 |
| *Coleonyx brevis* | Eublepharidae | 4.6 | Starostova, Kratochvil & Frynta, 2005 |
| *Coleonyx elegans* | Eublepharidae | 12.9 | Starostova, Kratochvil & Frynta, 2005 |
| *Coleonyx mitratus* | Eublepharidae | 12.95 | Starostova, Kratochvil & Frynta, 2005 |
| *Coleonyx variegatus* | Eublepharidae | 8.7 | Feldman et al., 2016 |
| *Coleonyx variegatus* | Eublepharidae | 4.7 | Starostova, Kratochvil & Frynta, 2005 |
| *Coluber constrictor* | Colubridae | 606.9 | Feldman et al., 2016 |
| *Coronella austriaca* | Colubridae | 66.5 | Feldman et al., 2016 |
| *Crocodylus niloticus* | Crocodylidae | 889320.97 | Hone et al., 2013 |
| *Crotalus durissus* | Viperidae | 2883 | Feldman et al., 2016 |
| *Crotalus viridis* | Viperidae | 2238.1 | Feldman et al., 2016 |
| *Crotaphytus collaris* | Crotaphytidae | 62.2 | Feldman et al., 2016 |
| *Ctenophorus reticulatus* | Agamidae | 42.4 | Feldman et al., 2016 |
| *Cyrtopodion heterocercum* | Gekkonidae | 3.1 | Feldman et al., 2016 |
| *Cyrtopodion scabrum* | Gekkonidae | 5.8 | Feldman et al., 2016 |
| *Darevskia praticola* | Lacertidae | 6.7 | Feldman et al., 2016 |
| *Darevskia rudis* | Lacertidae | 15.9 | Feldman et al., 2016 |
| *Darevskia uzzelli* | Lacertidae | 5.1 | Feldman et al., 2016 |
| *Darevskia valentini* | Lacertidae | 11.8 | Feldman et al., 2016 |
| *Deirochelys reticularia* | Emydidae | 2245.59 | Hone et al., 2013 |
| *Delma fraseri* | Pygopodidae | 8 | Feldman et al., 2016 |
| *Dendrophidion paucicarinatum* | Colubridae | 275.4 | Feldman et al., 2016 |
| *Dermochelys coriacea* | Dermochelyidae | 1030726.02 | Hone et al., 2013 |
| *Diporiphora bilineata* | Agamidae | 4.25 | Feldman et al., 2016 |
| *Dipsosaurus dorsalis* | Iguanidae | 159.7 | Feldman et al., 2016 |
| *Dolichophis caspius* | Colubridae | 1200.5 | Feldman et al., 2016 |
| *Dolichophis jugularis* | Colubridae | 1200.5 | Feldman et al., 2016 |
| *Dolichophis schmidti* | Colubridae | 386.6 | Feldman et al., 2016 |
| *Eirenis barani* | Colubridae | 19.4 | Feldman et al., 2016 |
| *Eirenis coronella* | Colubridae | 6 | Feldman et al., 2016 |
| *Eirenis decemlineatus* | Colubridae | 68 | Feldman et al., 2016 |
| *Eirenis eiselti* | Colubridae | 11.4 | Feldman et al., 2016 |
| *Eirenis levantinus* | Colubridae | 16.5 | Feldman et al., 2016 |
| *Eirenis modestus* | Colubridae | 32 | Feldman et al., 2016 |
| *Eirenis punctatolineatus* | Colubridae | 32 | Feldman et al., 2016 |
| *Eirenis rothii* | Colubridae | 8.2 | Feldman et al., 2016 |
| *Emys orbicularis* | Emydidae | 1232.64 | Hone et al., 2013 |
| *Eretmochelys imbricata* | Cheloniidae | 72591.53 | Hone et al., 2013 |
| *Erythrolamprus bizona* | Dipsadidae | 168.7 | Feldman et al., 2016 |
| *Erythrolamprus epinephelus* | Dipsadidae | 95.8 | Feldman et al., 2016 |
| *Erythrolamprus taeniurus* | Dipsadidae | 122.7 | Feldman et al., 2016 |
| *Eryx jaculus* | Boidae | 254.2 | Feldman et al., 2016 |
| *Eublepharis angramainyu* | Eublepharidae | 114.9 | Feldman et al., 2016 |
| *Eublepharis cf. fuscus* | Eublepharidae | 34.25 | Starostova, Kratochvil & Frynta, 2005 |
| *Eublepharius macularius* | Eublepharidae | 70.5 | Starostova, Kratochvil & Frynta, 2005 |
| *Eumeces schneideri* | Scincidae | 159.7 | Feldman et al., 2016 |
| *Gehyra variegata* | Gekkonidae | 7.4 | Feldman et al., 2016 |
| *Goniurosaurus araneus* | Eublepharidae | 22.25 | Starostova, Kratochvil & Frynta, 2005 |
| *Goniurosaurus luii* | Eublepharidae | 27.75 | Starostova, Kratochvil & Frynta, 2005 |
| *Gopherus polyphemus* | Testudinidae | 6929.19 | Hone et al., 2013 |
| *Graptemys flavimaculata* | Emydidae | 928.42 | Hone et al., 2013 |
| *Graptemys oculifera* | Emydidae | 1592.87 | Hone et al., 2013 |
| *Graptemys pulchra* | Emydidae | 3349 | Hone et al., 2013 |
| *Gyptemys muhlenbergii* | Emydidae | N.A. |  |
| *Hemidactylus turcicus* | Gekkonidae | 4.9 | Feldman et al., 2016 |
| *Hemitheconyx caudicinctus* | Eublepharidae | 57.45 | Starostova, Kratochvil & Frynta, 2005 |
| *Hemorrhois nummifer* | Colubridae | 217.2 | Feldman et al., 2016 |
| *Hemorrhois ravergieri* | Colubridae | 271.2 | Feldman et al., 2016 |
| *Heteronotia binoei* | Gekkonidae | 3.7 | Feldman et al., 2016 |
| *Hierophis viridiflavus* | Colubridae | 386.6 | Feldman et al., 2016 |
| *Holodactylus africanus* | Eublepharidae | 8.6 | Starostova, Kratochvil & Frynta, 2005 |
| *Iguana iguana* | Iguanidae | 8220.7 | Feldman et al., 2016 |
| *Kinosternon subrubrum* | Kinosternidae | 311.93 | Hone et al., 2013 |
| *Lacerta agilis* | Lacertidae | 33.6 | Feldman et al., 2016 |
| *Lacerta pamphylica* | Lacertidae | 39.1 | Feldman et al., 2016 |
| *Lacerta trilineata* | Lacertidae | 117.2 | Feldman et al., 2016 |
| *Lacerta viridis* | Lacertidae | 91.5 | Feldman et al., 2016 |
| *Lampropeltis triangulum* | Colubridae | 598.1 | Feldman et al., 2016 |
| *Laticauda colubrina* | Elapidae | 586.9 | Feldman et al., 2016 |
| *Leiolepis belliana* | Agamidae | 199.5 | Feldman et al., 2016 |
| *Lepidochelys kempii* | Cheloniidae | 38662.42 | Hone et al., 2013 |
| *Lepidochelys olivacea* | Cheloniidae | 37234.85 | Hone et al., 2013 |
| *Leptophis ahaetulla* | Colubridae | 918.7 | Feldman et al., 2016 |
| *Lialis burtonis* | Pygopodidae | 22.8 | Feldman et al., 2016 |
| *Lycodryas sp.* | Lamprophiidae | N.A. | Feldman et al., 2016 |
| *Macrochelys temminckii* | Chelydridae | 51330.64 | Hone et al., 2013 |
| *Macroprotodon cucullatus* | Colubridae | 25.7 | Feldman et al., 2016 |
| *Macrovipera lebetina* | Viperidae | 4769.8 | Feldman et al., 2016 |
| *Malpolon insignitus* | Lamprophiidae | 1186.5 | Feldman et al., 2016 |
| *Masticophis flagellum* | Colubridae | 1326.2 | Feldman et al., 2016 |
| *Mastigodryas boddaerti* | Colubridae | 331 | Feldman et al., 2016 |
| *Mauremys caspica* | Geoemydidae | 1902.12 | Hone et al., 2013 |
| *Mauremys rivulata* | Geoemydidae | N.A. |  |
| *Mesalina brevirostris* | Lacertidae | 5.1 | Feldman et al., 2016 |
| *Montivipera albizona* | Viperidae | 252.9 | Feldman et al., 2016 |
| *Montivipera wagneri* | Viperidae | 448.9 | Feldman et al., 2016 |
| *Montivipera xanthina* | Viperidae | 1118.3 | Feldman et al., 2016 |
| *Myriopholis macrorhyncha* | Leptotyphlopidae | 4.4 | Feldman et al., 2016 |
| *Naja kaouthia* | Elapidae | 1873.2 | Feldman et al., 2016 |
| *Naja naja* | Elapidae | 1683.5 | Feldman et al., 2016 |
| *Naja siamensis* | Elapidae | 782.2 | Feldman et al., 2016 |
| *Naja sumatrana* | Elapidae | 782.2 | Feldman et al., 2016 |
| *Natrix maura* | Colubridae | 135.8 | Feldman et al., 2016 |
| *Natrix natrix* | Colubridae | 2800 | Feldman et al., 2016 |
| *Natrix tesselata* | Colubridae | 283.8 | Feldman et al., 2016 |
| *Nerodia sipedon* | Colubridae | 1083 | Feldman et al., 2016 |
| *Nerodia taxispilota* | Colubridae | 1857 | Feldman et al., 2016 |
| *Opheodrys aestivus* | Colubridae | 170.5 | Feldman et al., 2016 |
| *Ophiomorus punctatissimus* | Scincidae | 2.5 | Feldman et al., 2016 |
| *Ophisops elegans* | Lacertidae | 8 | Feldman et al., 2016 |
| *Oxyuranus scutellatus* | Elapidae | 4665.5 | Feldman et al., 2016 |
| *Pantherophis guttatus* | Colubridae | 543.7 | Feldman et al., 2016 |
| *Parvilacerta parva* | Lacertidae | 5.6 | Feldman et al., 2016 |
| *Pelomedusa subrufa* | Pelomedusidae | 4740.97 | Hone et al., 2013 |
| *Phrynosoma mcallii* | Phrynosomatidae | 40 | Feldman et al., 2016 |
| *Platyceps collaris* | Colubridae | 117.2 | Feldman et al., 2016 |
| *Platyceps najadum* | Colubridae | 228.2 | Feldman et al., 2016 |
| *Platyceps ventromaculatus* | Colubridae | 210.8 | Feldman et al., 2016 |
| *Plestiodon fasciatus* | Scincidae | 13.2 | Feldman et al., 2016 |
| *Podarcis muralis* | Lacertidae | 11 | Feldman et al., 2016 |
| *Podarcis siculus* | Lacertidae | 16.7 | Feldman et al., 2016 |
| *Psammobates geometricus* | Testudinidae | 2012.95 | Hone et al., 2013 |
| *Psammodromus algirus* | Lacertidae | 18.4 | Feldman et al., 2016 |
| *Psammophilus blanfordanus* | Agamidae | 37.7 | Feldman et al., 2016 |
| *Pseudechis australis* | Elapidae | 2880.5 | Feldman et al., 2016 |
| *Pseudechis porphyriacus* | Elapidae | 1338.4 | Feldman et al., 2016 |
| *Pseudemys concinna* | Emydidae | 10279 | Hone et al., 2013 |
| *Python regius* | Pythonidae | 1610.6 | Feldman et al., 2016 |
| *Rhinechis scalaris* | Colubridae | 386.6 | Feldman et al., 2016 |
| *Rhynchocalamus melanocephalus* | Colubridae | 25.7 | Feldman et al., 2016 |
| *Sceloporus malachiticus* | Phrynosomatidae | 30.1 | Feldman et al., 2016 |
| *Spalerosophis diadema* | Colubridae | 681.3 | Feldman et al., 2016 |
| *Sphenodon punctatus* | Sphenodontidae | 1020 | Feldman et al., 2016 |
| *Spilotes pullatus* | Colubridae | 3030.1 | Feldman et al., 2016 |
| *Stellagama stellio* | Agamidae | 334.6 | Feldman et al., 2016 |
| *Sternotherus carinatus* | Kinosternidae | 928.43 | Hone et al., 2013 |
| *Sternotherus odoratus* | Kinosternidae | 472.23 | Hone et al., 2013 |
| *Suta suta* | Elapidae | 73.8 | Feldman et al., 2016 |
| *Telescopus fallax* | Colubridae | 186.2 | Feldman et al., 2016 |
| *Telescopus nigriceps* | Colubridae | 32 | Feldman et al., 2016 |
| *Terrapene carolina* | Emydidae | 1232.64 | Hone et al., 2013 |
| *Terrapene ornata* | Emydidae | 568.53 | Hone et al., 2013 |
| *Testudo graeca* | Testudinidae | 3668.77 | Hone et al., 2013 |
| *Thamnophis sauritus* | Colubridae | 299.2 | Feldman et al., 2016 |
| *Timon princeps* | Lacertidae | 72.7 | Feldman et al., 2016 |
| *Trachemys scripta* | Emydidae | 5991.26 | Hone et al., 2013 |
| *Trachylepis aurata* | Scincidae | 33.8 | Feldman et al., 2016 |
| *Trachylepis vittata* | Scincidae | 18.2 | Feldman et al., 2016 |
| *Trapelus lessonae* | Agamidae | N.A. | Feldman et al., 2016 |
| *Uma inornata* | Phrynosomatidae | 56.1 | Feldman et al., 2016 |
| *Urosaurus graciosus* | Phrynosomatidae | 11.2 | Feldman et al., 2016 |
| *Varanus exanthematicus* | Varanidae | 9995.9 | Feldman et al., 2016 |
| *Varanus griseus* | Varanidae | 5542.1 | Feldman et al., 2016 |
| *Varanus indicus* | Varanidae | 4352 | Feldman et al., 2016 |
| *Varanus komodoensis* | Varanidae | 102477.7 | Feldman et al., 2016 |
| *Vipera aspis* | Viperidae | 324.8 | Feldman et al., 2016 |
| *Vipera berus* | Viperidae | 227.4 | Feldman et al., 2016 |
| *Vipera eriwanensis* | Viperidae | 73.5 | Feldman et al., 2016 |
| *Walterinnesia morgani* | Elapidae | 368.2 | Feldman et al., 2016 |
| *Xerotyphlops vermicularis* | Typhlopidae | 39.6 | Feldman et al., 2016 |
| *Zamenis hohenackeri* | Colubridae | 58.4 | Feldman et al., 2016 |
| *Zamenis longissimus* | Colubridae | 681.3 | Feldman et al., 2016 |
| *Zootoca vivipara* | Lacertidae | 10.6 | Feldman et al., 2016 |
