# Supplementary material for: Hematological convergence between Mesozoic marine reptiles (Sauropterygia) and extant aquatic amniotes elucidates diving adaptations in plesiosaurs
Source: PeerJ. 2019 Nov 19;7:e8022. doi: 10.7717/peerj.8022 (PMC6873879; doi:10.7717/peerj.8022)
Supplement: Supplemental Information 9 — When studies presented data of several individuals or populations those were averaged before the inclusion into the table. Names listed have been checked to comply with current taxonomic nomenclature and for this reason might deviate from the ones listed in the referenced literature. *Only values for adults were used although data for juveniles were given; **Study only investigated juveniles ***Values from wild caught individuals were used. [file peerj-07-8022-s009.docx]

| **Species** | **Family** | **Ø RBC Volume** | **Reference** |
| --- | --- | --- | --- |
| **Terrestrial Mammalia** |  |  |  |
| *Antilocapra americana* | Antilocapridae | 41 | Hawkey, 1975 |
| *Antilope cervicapra* | Bovidae | 45 | Hawkey, 1975 |
| *Aotus trivirgatus* | Aotidae | 86 | Hawkey, 1975 |
| *Ateles paniscus* | Atelidae | 79 | Hawkey, 1975 |
| *Axis axis* | Cervidae | 24 | Hawkey, 1975 |
| *Bos taurus* | Bovidae | 58 | Gregory, 2000 |
| *Boselaphus tragocamelus* | Bovidae | 59 | Hawkey, 1975 |
| *Callithrix jacchus* | Callitrichidae | 71 | Hawkey, 1975 |
| *Camelus bactrianus* | Camelidae | 27 | Hawkey, 1975 |
| *Camelus dromedarius* | Camelidae | 40 | Hawkey, 1975 |
| *Canis aureus* | Canidae | 70 | Gregory, 2000 |
| *Canis familiaris* | Canidae | 66 | Gregory, 2000 |
| *Capra hircus* | Bovidae | 18 | Gregory, 2000 |
| *Cavia porcellus* | Caviidae | 77 | Gregory, 2000 |
| *Cebus capucinus* | Cebidae | 86 | Hawkey, 1975 |
| *Ceratotherium simum* | Rhinocerotidae | 65 | Hawkey, 1975 |
| *Cervus nippon* | Cervidae | 35 | Hawkey, 1975 |
| *Connochaetes gnou* | Bovidae | 40 | Hawkey, 1975 |
| *Dama dama* | Cervidae | 46 | Hawkey, 1975 |
| *Dendrolagus goodfellowi* | Macropodidae | 81 | Hawkey, 1975 |
| *Didelphis aurita* | Didelphidae | 79 | Gregory, 2000 |
| *Eira barbara* | Mustelidae | 64 | Hawkey, 1975 |
| *Elephas maximus* | Elephantidae | 125 | Hawkey, 1975 |
| *Equus caballus* | Equidae | 52 | Hawkey, 1975 |
| *Equus hemionus* | Equidae | 68 | Hawkey, 1975 |
| *Equus quagga* | Equidae | 54 | Hawkey, 1975 |
| *Galago senegalensis* | Galagidae | 70 | Haines et al., 1971 |
| *Giraffa camelopardalis* | Giraffidae | 39 | Hawkey, 1975 |
| *Herpestes urva* | Herpestidae | 42 | Hawkey, 1975 |
| *Homo sapiens* | Hominidae | 90 | Gregory, 2000 |
| *Hoolock hoolock* | Hylobatidae | 82 | Hawkey, 1975 |
| *Hyaena hyaena* | Hyaenidae | 61 | Hawkey, 1975 |
| *Hydrochoerus hydrochaeris* | Caviidae | 114 | Hawkey, 1975 |
| *Kobus ellipsiprymnus* | Bovidae | 51 | Hawkey, 1975 |
| *Lagostomus maximus* | Chinchillidae | 85 | Hawkey, 1975 |
| *Lama glama* | Camelidae | 28 | Hawkey, 1975 |
| *Lemur catta* | Lemuridae | 78 | Hawkey, 1975 |
| *Loxodonta africana* | Elephantidae | 141 | Gregory, 2000 |
| *Lycaon pictus* | Canidae | 68 | Hawkey, 1978 |
| *Macaca sylvanus* | Cercopithecidae | 82 | Hawkey, 1975 |
| *Macropus rufogriseus* | Macropodidae | 90 | Hawkey, 1975 |
| *Marmota monax* | Sciuridae | 66 | Gregory, 2000 |
| *Mesocricetus auratus* | Cricetidae | 62 | Hawkey, 1975 |
| *Mus musculus* | Muridae | 48 | Gregory, 2000 |
| *Mustela vison* | Mustelidae | 68 | Kubin & Mason, 1948 |
| *Myocastor coypus* | Echimyidae | 117 | Hawkey, 1975 |
| *Myrmecophaga tridactyla* | Myrmecophagidae | 162 | Hawkey, 1975 |
| *Nasua nasua* | Procyonidae | 59 | Hawkey, 1975 |
| *Oryctolagus cuniculus* | Leporidae | 61 | Gregory, 2000 |
| *Otocyon megalotis* | Canidae | 71 | Hawkey, 1977 |
| *Otolemur crassicaudatus* | Galagidae | 55 | Hawkey, 1975 |
| *Ovis aries* | Bovidae | 32 | Gregory, 2000 |
| *Pan troglodytes* | Hominidae | 82 | Gregory, 2000 |
| *Panthera leo* | Felidae | 53 | Hawkey, 1975 |
| *Panthera tigris* | Felidae | 60 | Hawkey, 1975 |
| *Papio anubis* | Cercopithecidae | 89 | Hawkey, 1975 |
| *Papio hamadryas* | Cercopithecidae | 91 | Hawkey, 1975 |
| *Paradoxurus hermaphroditus* | Viverridae | 40 | Hawkey, 1975 |
| *Phacochoerus aethiopicus* | Suidae | 63 | Hawkey, 1975 |
| *Potamochoerus porcus* | Suidae | 56 | Hawkey, 1975 |
| *Potos flavus* | Procyonidae | 55 | Gregory, 2000 |
| *Procyon lotor* | Procyonidae | 60 | Hawkey, 1975 |
| *Puma concolor* | Felidae | 48 | Hawkey, 1975 |
| *Rangifer tarandus* | Cervidae | 48 | Hawkey, 1975 |
| *Rattus rattus* | Muridae | 41 | Gregory, 2000 |
| *Rhinoceros unicornis* | Rhinocerotidae | 65 | Hawkey, 1975 |
| *Saiga tatarica* | Bovidae | 59 | Hawkey, 1975 |
| *Sorex araneus* | Soricidae | 31 | Gregory, 2000 |
| *Speothos venaticus* | Canidae | 85 | Hawkey, 1979 |
| *Strepsiceros strepsiceros* | Bovidae | 54 | Hawkey, 1975 |
| *Sus scrofa* | Suidae | 58 | Gregory, 2000 |
| *Tachyglossus aculeatus* | Tachyglossidae | 61 | Hawkey, 1975 |
| *Tapirus indicus* | Tapiridae | 62 | Hawkey, 1975 |
| *Tapirus terrestris* | Tapiridae | 51 | Hawkey, 1975 |
| *Tragulus javanicus* | Tragulidae | 6 | Gregory, 2000 |
| *Tremarctos ornatus* | Ursidae | 48 | Hawkey, 1975 |
| *Tupaia glis* | Tupaiidae | 67 | Hunt, 1967 |
| *Ursus arctos* | Ursidae | 68 | Hawkey, 1975 |
| *Ursus maritimus* | Ursidae | 69 | Hawkey, 1975 |
| *Ursus thibetanus* | Ursidae | 76 | Hawkey, 1975 |
| *Vombatus ursinus* | Vombatidae | 74 | Hawkey, 1975 |
| *Vulpes vulpes* | Canidae | 53 | Gregory, 2000 |
|  |  |  |  |
| **Cetacea** |  |  |  |
| *Balaena mysticetus* | Balaenidae | 178 | Castellini et al., 2006 |
| *Cephalorynchus commersoni* | Delphinidae | 105 | Hedrick & Duffield, 1990 |
| *Delphinapterus leucas* | Monodontidae | 132 | MacNeill, 1975 |
| *Eschrichtius robustus* | Eschrichtiidae | 133 | Lenfant, 1969 |
| *Globicephala macrorhynchos* | Delphinidae | 119 | MacNeill, 1975 |
| *Globicephala melas* | Delphinidae | 123 | MacNeill, 1975 |
| *Inia geoffrensis* | Iniidae | 106 | MacNeill, 1975 |
| *Lagenorhynchus obliquidens* | Delphinidae | 96 | MacNeill, 1975 |
| *Lipotes vexillifer* | Lipotidae | 127 | Guan & Chen, 1989 |
| *Monodon monoceros* | Monodontidae | 164 | MacNeill, 1975 |
| *Orcinus orca* | Delphinidae | 112 | MacNeill, 1975 |
| *Phocoena phocoena* | Phocoenidae | 100 | MacNeill, 1975 |
| *Phocoenoides dalli* | Phocoenidae | 96 | MacNeill, 1975 |
| *Tursiops truncatus* | Delphinidae | 107 | MacNeill, 1975 |
|  |  |  |  |
| **Pinnipedia** |  |  |  |
| *Arctocephalus australis** | Otariidae | 100 | Seguel et al., 2016 |
| *Eumetopias jubatus*** | Otariidae | 108 | Richmond et al., 2005 |
| *Histriophoca fasciata* | Phocidae | 150 | Wickham et al., 1989 |
| *Leptonychotes weddelli* | Phocidae | 155 | Castellini et al., 2006 |
| *Mirounga* sp. | Phocidae | 176 | Wickham et al., 1989 |
| *Monachus schauinslandi*** | Phocidae | 151 | Banish & Gilmartin, 1988 |
| *Neophoca cinerea* | Otariidae | 103 | Wickham et al., 1989 |
| *Pagophilus groenlandicus** | Phocidae | 131 | Geraci, 1971 |
| *Phoca vitulina* | Phocidae | 105 | Wickham et al., 1989 |
| *Pusa hispida** | Phocidae | 135 | Geraci & Smith, 1975 |
| *Pusa sibirica* | Phocidae | 122 | Hawkey, 1975 |
| *Zalophus californianus* | Otariidae | 97 | Hedrick and Duffield |
|  |  |  |  |
| **Sea otter** |  |  |  |
| *Enhydra lutris**** | Mustelidae | 113 | Wickham et al., 1990 |
|  |  |  |  |
| **Sirenia** |  |  |  |
| *Trichechus manatus* | Trichechidae | 133 | Medway et al., 1982 |
