# Supplementary material for: Hematological convergence between Mesozoic marine reptiles (Sauropterygia) and extant aquatic amniotes elucidates diving adaptations in plesiosaurs
Source: PeerJ. 2019 Nov 19;7:e8022. doi: 10.7717/peerj.8022 (PMC6873879; doi:10.7717/peerj.8022)
Supplement: Supplemental Information 10 — When studies presented data of several individuals or populations those were averaged before the inclusion into the table. Names listed have been checked to comply with current taxonomic nomenclature and for this reason might deviate from the ones listed in the referenced literature. [file peerj-07-8022-s010.docx]

| **Species** | **Family** | **Ø RBC Volume (µm^3^)** | **Reference** |
| --- | --- | --- | --- |
| **Non-sphenisciform birds** |  |  |  |
| *Alopochen aegyptiaca* | Anatidae | 138 | Fourie & Hattingh, 1983 |
| *Amadina erythrocephala* | Estrildidae | 120 | Fourie & Hattingh, 1983 |
| *Anas erythroryncha* | Anatidae | 127 | Fourie & Hattingh, 1983 |
| *Anas undulata* | Anatidae | 129 | Fourie & Hattingh, 1983 |
| *Anthropoides paradisea* | Gruidae | 160 | Fourie & Hattingh, 1983 |
| *Aquila rapax* | Accipitridae | 141 | Fourie & Hattingh, 1983 |
| *Ardea cinerea* | Ardeidae | 161 | Fourie & Hattingh, 1983 |
| *Aythya ferina* | Anatidae | 134 | Balasch et al., 1974 |
| *Bubo africanus* | Strigidae | 160 | Fourie & Hattingh, 1983 |
| *Columba guinea* | Columbidae | 125 | Fourie & Hattingh, 1983 |
| *Columba livia* | Columbidae | 147 | Fourie & Hattingh, 1983 |
| *Corvus albus* | Corvidae | 152 | Fourie & Hattingh, 1983 |
| *Corvus corax* | Corvidae | 128 | Balasch et al., 1974 |
| *Coturnix coturnix* | Phasianidae | 66 | Fourie & Hattingh, 1983 |
| *Falco biarmicus* | Falconidae | 133 | Fourie & Hattingh, 1983 |
| *Falco rupicoloides* | Falconidae | 158 | Fourie & Hattingh, 1983 |
| *Francolinus afer* | Phasianidae | 100 | Fourie & Hattingh, 1983 |
| *Francolinus natalensis* | Phasianidae | 95 | Fourie & Hattingh, 1983 |
| *Gallus domesticus* | Phasianidae | 127 | Fourie & Hattingh, 1983 |
| *Larus argentatus* | Laridae | 145 | Balasch et al., 1974 |
| *Larus ridibundus* | Laridae | 144 | Balasch et al., 1974 |
| *Macronectes giganteus* | Procellariidae | 175 | Block & Murrish, 1974 |
| *Morus bassanus* | Sulidae | 156 | Balasch et al., 1974 |
| *Numida meleagris* | Numididae | 140 | Fourie & Hattingh, 1983 |
| *Oxyura punctata* | Anatidae | 121 | Fourie & Hattingh, 1983 |
| *Passer melanurus* | Passeridae | 121 | Fourie & Hattingh, 1983 |
| *Pelecanus occidentalis* | Pelecanidae | 160 | Balasch et al., 1974 |
| *Phalacrocorax atriceps* | Phalacrocoracidae | 228 | Block & Murrish, 1974 |
| *Phalacrocorax carbo* | Phalacrocoracidae | 151 | Balasch et al., 1974 |
| *Plectropterus gambensis* | Anatidae | 153 | Fourie & Hattingh, 1983 |
| *Pycnonotus barbatus* | Pycnonotidae | 150 | Fourie & Hattingh, 1983 |
| *Stercorarius maccormicki* | Stercocariidae | 143 | Block & Murrish, 1974 |
| *Streptopelia senegalensis* | Columbidae | 162 | Fourie & Hattingh, 1983 |
| *Turtur tympanistria* | Columbidae | 108 | Fourie & Hattingh, 1983 |
| *Tyto alba* | Tytonidae | 170 | Fourie & Hattingh, 1983 |
| *Tyto capensis* | Tytonidae | 183 | Fourie & Hattingh, 1983 |
|  |  |  |  |
| **Sphenisciformes** |  |  |  |
| *Aptenodytes patagonicus* | Spheniscidae | 209 | Fayolle et al., 2000 |
| *Eudyptula minor* | Spheniscidae | 229 | Nicol et al., 1988 |
| *Pygoscelis adelie* | Spheniscidae | 239 | Block & Murrish, 1974 |
| *Pygoscelis antarctica* | Spheniscidae | 224 | Block & Murrish, 1974 |
| *Pygoscelis papua* | Spheniscidae | 284 | Block & Murrish, 1974 |
| *Spheniscus magellanicus* | Spheniscidae | 253 | Baldassin et al., 2013 |
