# Supplementary material for: Hematological convergence between Mesozoic marine reptiles (Sauropterygia) and extant aquatic amniotes elucidates diving adaptations in plesiosaurs
Source: PeerJ. 2019 Nov 19;7:e8022. doi: 10.7717/peerj.8022 (PMC6873879; doi:10.7717/peerj.8022)
Supplement: Supplemental Information 11 [file peerj-07-8022-s011.docx]

**Phylogenetic framework for RBC parameter estimation**

BEGIN TAXA;

TITLE Untitled_Block_of_Taxa;

DIMENSIONS NTAX=27;

TAXLABELS

Xenopus Lithobates Alligator Anas Lepus_californicus Oryctolagus_cuniculus Rattus Sciurus Columba_livia Varanus_komodoensis Varanus_exanthematicus Trachemys_scripta Neovison_vison Didelphis_virginiana Neusticosaurus_edwardsii Anarosaurus_heterodontus Nothosaurus Pistosaurus_longaevus Rhaeticosaurus_mertensi Plesiosaurus_dolichodeirus Pliosaurus Elasmosauridae Polycotylus_latipinnus Cryptoclidus_eurymerus Cymatosaurus Neusticosaurus_pusillus Neusticosaurus_peyeri

;

END;

BEGIN TREES;

TRANSLATE

1 Xenopus,

2 Lithobates,

3 Alligator,

4 Anas,

5 Lepus_californicus,

6 Oryctolagus_cuniculus,

7 Rattus,

8 Sciurus,

9 Columba_livia,

10 Varanus_komodoensis,

11 Varanus_exanthematicus,

12 Trachemys_scripta,

13 Neovison_vison,

14 Didelphis_virginiana,

15 Neusticosaurus_edwardsii,

16 Anarosaurus_heterodontus,

17 Nothosaurus,

18 Pistosaurus_longaevus,

19 Rhaeticosaurus_mertensi,

20 Plesiosaurus_dolichodeirus,

21 Pliosaurus,

22 Elasmosauridae,

23 Polycotylus,

24 Cryptoclidus,

25 Cymatosaurus,

26 Neusticosaurus_peyeri,

27 Neusticosaurus_pusillus;

TREE 'Edited, based on Edited, based on Default Symmetrical Tree++' = (((((3:249.1,(9:122.0,4:122.0):127.1):4.9,12:254):31,((10:17.8,11:17.8):235.2,((16:1,(27:1,(15:3,26:1):2):6):1,(17:1,(25:2,(18:1,((19:2,21:37):5,(20:4,(22:52,(23:89,24:6):11):17):8):39):1):1):1):6):32):37.4,(14:163.7,(13:96.2,((5:15.0,6:15.0):71.1,(7:74.3,8:74.3):11.8):10.1):67.5):158.7):36.7,(1:198.6,2:198.6):160.5):1.0;

END;
