# Supplementary material for: Hematological convergence between Mesozoic marine reptiles (Sauropterygia) and extant aquatic amniotes elucidates diving adaptations in plesiosaurs
Source: PeerJ. 2019 Nov 19;7:e8022. doi: 10.7717/peerj.8022 (PMC6873879; doi:10.7717/peerj.8022)
Supplement: Supplemental Information 12 [file peerj-07-8022-s012.docx]

**References cited in supplements**

Arikan, H. & Cicek, K. (2014). Haematology of amphibians and reptiles: a review. *North-Western Journal of Zoology* 10: 190-209.

Balasch, J., Palomeque, J., Palacios, L., Musquera, S., & Jimenez, M. (1974). Hematological values of some great flying and aquatic-diving birds. *Comparative Biochemistry and Physiology. A, Comparative Physiology* 49: 137-145.

Baldassin, P., Werneck, M. R., & Torres, F. (2013) First hemogram during *Spheniscus magellanicus* rehabilitation program. Conference abstract, *8^th^ International Penguin Conference, Bristol.*

Banish, L. D., & Gilmartin, W. G. (1988). Hematology and serum chemistry of the young Hawaiian monk seal (*Monachus schauinslandi*). *Journal of Wildlife Diseases* 24: 225-230.

Block, G. A., & Murrish, D. E. (1974). Viscous properties of bird blood at low temperatures. *Antarctic Journal of the United States* 9: 98-99.

Castellini, M., Elsner, R., Baskurt, O. K., Wenby, R. B., & Meiselman, H. J. (2006). Blood rheology of Weddell seals and bowhead whales. *Biorheology* 43: 57-69.

Duermit, L. 2007. *Caretta caretta* (On-line), Animal Diversity Web. Accessed July 18, 2019 at https://animaldiversity.org/accounts/Caretta_caretta/

Fayolle, C., Leray, C., Ohlmann, P., Gutbier, G., Cazenave, J. P., Gachet, C., & Groscolas, R. (2000). Lipid composition of erythrocytes and thrombocytes of a subantarctic seabird, the king penguin. Lipids 35: 453-459.

Feldman, A., Sabath, N., Pyron, R. A., Mayrose, I., & Meiri, S. (2016). Body sizes and diversification rates of lizards, snakes, amphisbaenians and the tuatara. *Global Ecology and Biogeography* 25: 187-197.

Fourie, F. L. R., & Hattingh, J. (1983). Comparative haematology of some South African birds. *Comparative Biochemistry and Physiology Part A: Physiology* 74: 443-448.

Frair, W. (1977). Sea turtle red blood cell parameters correlated with carapace lengths. *Comparative Biochemistry and Physiology Part A: Physiology* 56: 467-472.

Frýdlová, P., Hnizdo, J., Chylikova, L., Šimková, O., Cikanova, V., Velenský, P., & Frynta, D. (2013). Morphological characteristics of blood cells in monitor lizards: is erythrocyte size linked to actual body size?. *Integrative Zoology* 8: 39-45.

Geraci, J. R. (1971). Functional hematology of the harp seal *Pagophilus groenlandicus*. *Physiological Zoology* 44: 162-170.

Geraci, J. R., & Smith, T. G. (1975). Functional hematology of ringed seals (*Phoca hispida*) in the Canadian Arctic. *Journal of the Fisheries Board of Canada*, 32: 2559-2564.

Gregory, T. R. (2000). Nucleotypic effects without nuclei: genome size and erythrocyte size in mammals. *Genome* 43: 895-901.

Guan, Z., & Chen, D. (1989). Hematology of the Baiji, *Lipotes vexillifer*. *Occasional Papers of the IUCN SSC* 3: 114-118.

Haines, D. E., Holmes, K. R., & Brett, I. J. (1971). The hemogram of the colonized lesser bushbaby (*Galago senegalensis*). *Folia Primatologica*, 14: 95-100.

Hartman, F. A., & Lessler, M. A. (1964). Erythrocyte measurements in fishes amphibia, and reptiles. *The Biological Bulletin* 126: 83-88.

Hedrick, M. S., & Duffield, D. A. (1991). Haematological and rheological characteristics of blood in seven marine mammal species: physiological implications for diving behaviour. *Journal of Zoology* 225: 273-283.

Hone, D & O'Gorman, E.J. (2013): Body Size datasets for PLOS ONE paper doi:10.1371. figshare. http://dx.doi.org/10.6084/m9.figshare.627530 Retrieved July 09, 2015**.**

Hunt, R. D. (1967). The hemogram of the tree shrew (*Tupaia glis*). *Folia Primatologica* 7: 34-36.

Kubin, R., & Mason, M. M. (1948). Normal blood and urine values for mink. *The Cornell veterinarian*, 38. 79-85.

Lenfant, C. (1969). Physiological properties of blood of marine mammals in H. T. Anderson (ed.). *The Biology of Marine Mammals*. Academic Press, New York, pp. 95-116, cited in Priddel, D., & Wheeler, R. (1998). Hematology and blood chemistry of a Bryde's whale, *Balaenoptera edeni*, entrapped in the Manning River, New South Wales, Australia. *Marine Mammal Science* 14: 72-81.

Medway, W., Black, D. J., & Rathbun, G. B. (1982). Hematology of the West Indian manatee (*Trichechus manatus*). *Veterinary Clinical Pathology* 11: 11-15.

Nicol, S. C., Melrose, W., &Stahel, C. D. (1988). Haematology and metabolism of the blood of the little penguin, *Eudyptula minor*. *Comparative biochemistry and Physiology. A, Comparative Physiology* 89: 383-386.

O’Dwyer, L. H., Moco, T. C., & da Silva, R. J. (2004). Description of the gamonts of a small species of *Hepatozoon* sp. (Apicomplexa, Hepatozoidae) found in *Crotalus durissus terrificus* (Serpentes, Viperidae). *Parasitology Research* 92: 110-112.

Richmond, J. P., Burns, J. M., Rea, L. D., & Mashburn, K. L. (2005). Postnatal ontogeny of erythropoietin and hematology in free-ranging Steller sea lions (*Eumetopias jubatus*). *General and Comparative Endocrinology* 141: 240-247.

Saint Girons, M. C. (1970). Morphology of the circulating blood cells in Gans, C. and Parsons, T. S. (eds.) Biology of the Reptilia. Volume 3. Morphology C. Academic Press, London and New York*,* pp. 73-91.

Seguel, M., Muñoz, F., Keenan, A., Perez-Venegas, D. J., DeRango, E., Paves, H., ... & Müller, A. (2016). Hematology, serum chemistry, and early hematologic changes in free-ranging South American fur seals (*Arctocephalus australis*) at Guafo Island, Chilean Patagonia. *Journal of Wildlife Diseases* 52: 663-668.

Sevi̇nç, M., Uğurtaş, İ. H., & Yildirimhan, H. S. (2000). Erythrocyte measurements in *Lacerta rudis* (Reptilia, Lacertidae). *Turkish Journal of Zoology*, 24: 207-210.

Sloboda, M., Kamler, M., Bulantová, J., Votýpka, J., & Modrý, D. (2007). A new species of *Hepatozoon* (Apicomplexa: Adeleorina) from *Python regius* (Serpentes: Pythonidae) and its experimental transmission by a mosquito vector. *Journal of Parasitology* 93:1189-1198.

Starostová, Z., Kratochvíl, L., & Frynta, D. (2005). Dwarf and giant geckos from the cellular perspective: the bigger the animal, the bigger its erythrocytes?. *Functional Ecology* 19: 744-749.

Wickham, L. L., Costa, D. P., & Elsner, R. (1990). Blood rheology of captive and free-ranging northern elephant seals and sea otters. *Canadian Journal of Zoology* 68: 375-380.

Wickham, L. L., Elsner, R., White, F. C., & Cornell, L. H. (1989). Blood viscosity in phocid seals: possible adaptations to diving. *Journal of Comparative Physiology B* 159: 153-158.
